# Distinguishing neutral from deleterious mutations in growing populations

**DOI:** 10.3389/fgene.2014.00007

**Published:** 2014-01-31

**Authors:** Greg B. Ewing, Jeffrey D. Jensen

**Affiliations:** School of Life Sciences, Ecole Polytechnique Federale de Lausanne, Lausanne Switzerland, Swiss Institute of BioinformaticsLausanne, Switzerland

**Keywords:** population genetics, population growth, distribution of fitness effects, disease-causing mutations, coalescent theory

The identification of disease causing rare variants is becoming possible with the advent of next generation sequencing (Nejentsev et al., 2009; Calvo et al., 2010; Johansen et al., 2010). Recently Kim and Schuster (2013) explored this task with large numbers of publicly available mitochondrial genomes, in an attempt to disentangle selective from demographic effects. The recent rapid population expansion of humans is expected to give rise to an excess of rare variants both neutral and deleterious (Keinan and Clark, 2012) and is thus expected to make the detection of these rare disease causing variants more difficult. Kim and Schuster (2013) first infer the demographic history of the population, finding support for a model of population structure and expansion, as supported by previous studies (Gutenkunst et al., 2009; Gravel et al., 2011) but, with a lower growth rate. Forward simulations are used under this demographic model to determine the maximum relative frequency that negatively selected alleles may be expected to reach in each of the considered sub-populations. It should be pointed out that their criteria was simply the largest frequency observed across 10,000 simulations, and is thus expected to be conservative. It is found that most of the currently known and suspected disease rare variants had frequencies below the threshold and account for a small portion of the total rare variants. Surprisingly however, a number of disease rare variants (>5%) showed frequencies above the threshold which suggests other forces acting on these SNVs. Perhaps the more interesting result is the sample sizes required to provide reasonable power to investigate disease causing rare variants. Although high (2400–7400 per subpopulation), such sample sizes seem possible within the next decade given the reduced cost and higher throughput of sequencing technologies.

An intuitive understanding of the underlying dynamics of the evolution of rare variants can be gained by considering the coalescent trees that are likely under models of population growth. Population growth results in trees where coalescent events are rare in the recent past and tend toward a more star like topology (Wakeley, 2009), owing to the relationship between population size and rate of coalescence. This results in long terminal branches relative to internal branches—resulting in an increased number of rare variants and a decreased number of intermediate frequency variants relative to the equilibrium neutral model. Under such a topology, singletons become more common and intermediate frequency variants are reduced in prevalence. The problem of detecting rare disease alleles is thus effectively more difficult, as the needles are now in a larger haystack. Because of long terminal branches, we would expect neutral singleton SNPs on shared haplotypes. Additionally contributing to this challenge, sequencing errors make it difficult to call rare variants, and thus the mutations of interest may be excluded from analysis depending on their frequency in the population. In Kim and Schuster (2013), data sets are split into data with and without singletons for analysis and comparison.

The picture with selection is less intuitive, but as population sizes increase, drift becomes less dominant and selection is more effective. Gazave et al. (2013) provides valuable insight into low frequency deleterious mutations under population growth. Their findings showed that while the number of deleterious mutations per individual increased, the mean effect decreased. In their study, mutations are not independent and more importantly, are considered as a distribution of fitness effects (DFE). Within this context, Gazave et al. (2013) argued that selection acted more efficiently on strongly deleterious mutations reducing their frequency, while mildly deleterious mutations were more prevalent. Even though Kim and Schuster (2013) assumes non-interfering independent mutations, simulations carried out with a higher rate of population growth showed a lower frequency of deleterious mutations compared with weaker growth models. This suggests that under expanding populations, disease risk is distributed over a larger number of weakly deleterious mutations as compared to equilibrium populations.

A contrasting point between these studies is the assumption of independence of the deleterious mutations in Kim and Schuster (2013) when calculating upper frequency bounds. It has been suggested that the high frequency of some rare disease mutations in human populations is due to hitchhiking with a nearby beneficial mutation. This is one explanation that may explain why some disease variants have frequency above the conservative threshold. Indeed, perhaps the most important effect in growing populations is the dependence on DFE when considering complete haplotype fitness. Weak beneficial mutations will have a better chance of establishing in the population as the results of Gazave et al. (2013) suggest. These more prevalent beneficial mutations would then help pull deleterious mutations to higher frequency than would otherwise be expected. Conversely, as the rate of recombination increases, Hill-Robertson effects (Hill and Robertson, 1966) will be minimized providing a mechanisms for nearly neutral mutations to escape from strongly deleterious mutations over time. It should be clear that the form of the distribution around neutrality will have an impact on the expected numbers of deleterious mutations in any individual (Figure 1). Further investigation of the effects and sensitivity of the DFE in expanding populations would be an interesting contribution to the field.

**Figure 1:**
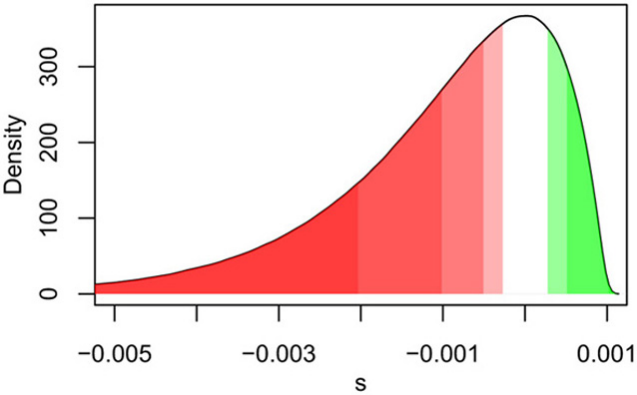
**A visual representation of the effect of increasing population size (*N*_*e*_) on the fraction of deleterious, neutral, and beneficial mutations, for a given potential realization of the distribution of fitness effects (DFE)**. As shown, the effective fraction of neutral mutations decreases with increasing *N*_*e*_ with the darkest shades representing the proportion of deleterious (red) and beneficial (green) mutations for *N*_*e*_ = 500, and increasingly lighter shades for (*N*_*e*_ = 1000, 2000, and 4000). The white shading centered around 0 represents mutations that are effectively neutral under all four population sizes.

Finding disease causing rare variants or even risk factors in humans remains difficult, in part, owing to recent expansion. Larger sample sizes, while considering nonindependent mutations drawn from the DFE, is a promising way forward. Currently, the sample sizes required seem huge, but continued advances in sequencing technology they are increasingly feasible - indeed, we can expect to have samples sizes comparable to effective population size in the near future. However, the coalescent traditionally requires that sample size be much smaller than effective population size and extensions or behavior of the coalescent with violations would need to be considered. Wakeley and Takahashi (2003) investigated the even more extreme case of sample size exceeding effective population size, with the result that rare mutations are even more prevalent and that mutation rate and effective population size can be separately estimated. Thus, continued theoretical development combined with extensive on-going sequencing efforts may indeed help to differentiate the fraction of new and segregating deleterious mutations in human populations.

## References

[B1] Calvo S. E. Tucker E. J. Compton A. G. Kirby D. M. Crawford G. Burtt N. P. (2010). High-throughput, pooled sequencing identifies mutations in NUBPL and FOXRED1 in human complex i deficiency. Nat. Genet. 42, 851–858 10.1038/ng.659 20818383 PMC2977978

[B2] Gazave E. Chang D. Clark A. G. Keinan A. (2013). Population growth inflates the per-individual number of deleterious mutations and reduces their mean effect. Genetics 195, 969–978 10.1534/genetics.113.153973 23979573 PMC3813877

[B3] Gravel S. Henn B. M. Gutenkunst R. N. Indap A. R. Marth G. T. Clark A. G. (2011). Demographic history and rare allele sharing among human populations. Proc. Natl. Acad. Sci. U.S.A. 108, 11983–11988 10.1073/pnas.1019276108 21730125 PMC3142009

[B4] Gutenkunst R. N. Hernandez R. D. Williamson S. H. Bustamante C. D. (2009). Inferring the joint demographic history of multiple populations from multidimensional SNP frequency data. PLoS Genet. 5:e1000695 10.1371/journal.pgen.1000695 19851460 PMC2760211

[B5] Hill W. G. Robertson A. (1966). The effect of linkage on limits to artificial selection. Genet. Res. 8, 269–294 10.1017/S0016672300010156 5980116

[B6] Johansen C. T. Wang J. Lanktree M. B. Cao H. McIntyre A. D. Ban M. R. (2010). Excess of rare variants in genes identified by genome-wide association study of hypertriglyceridemia. Nat. Genet. 42, 684–687 10.1038/ng.628 20657596 PMC3017369

[B7] Keinan A. Clark A. G. (2012). Recent explosive human population growth has resulted in an excess of rare genetic variants. Science 336, 740–743 10.1126/science.1217283 22582263 PMC3586590

[B8] Kim H. L. Schuster S. C. (2013). Poor man's 1000 genome project: recent human population expansion confounds the detection of disease alleles in 7,098 complete mitochondrial genomes. Front. Evol. Popul. Genet. 4:13 10.3389/fgene.2013.00013 23450075 PMC3584485

[B9] Nejentsev S. Walker N. Riches D. Egholm M. Todd J. A. (2009). Rare variants of IFIH1, a gene implicated in antiviral responses, protect against type 1 diabetes. Science 324, 387–389 10.1126/science.1167728 19264985 PMC2707798

[B10] Wakeley J. (2009). Coalescent Theory: an Introduction. Greenwood Village, Colo: Roberts & Co. Publishers

[B11] Wakeley J. Takahashi T. (2003). Gene genealogies when the sample size exceeds the effective size of the population. Mol. Biol. Evol. 20, 208–213 10.1093/molbev/msg024 12598687

